# Study of Vertical Phototransistors Based on Integration of Inorganic Transistors and Organic Photodiodes

**DOI:** 10.3390/mi15111397

**Published:** 2024-11-20

**Authors:** Jui-Fen Chang, Ying-You Lin, Yu-Ming Li

**Affiliations:** Department of Optics and Photonics, National Central University, Zhongli 320317, Taiwan; l.y.y111226061@gmail.com (Y.-Y.L.); james80779771@gmail.com (Y.-M.L.)

**Keywords:** inorganic transistors, organic photodiodes, vertical phototransistors

## Abstract

We investigate the inorganic/organic hybrid vertical phototransistor (VPT) by integrating an atomic layer deposition-processed ZnO (ALD-ZnO) transistor with a prototype poly(3-hexylthiophene):[6,6]-phenyl-C_61_-butyric acid methyl ester (P3HT:PC_61_BM) blend organic photodiode (OPD) based on an encapsulated source electrode geometry, and discuss the device mechanism. Our preliminary studies on reference P3HT:PC_61_BM OPDs show non-ohmic electron injection between the ALD-ZnO and P3HT:PC_61_BM layers. However, the ALD-ZnO layer enables the accumulation of photogenerated holes under negative bias, which facilitates electron injection upon illumination and thereby enhances the external quantum efficiency (*EQE*). This mechanism underpins the photoresponse in the VPT. Furthermore, we demonstrate that the gate field in the VPT effectively modulates electron injection from the ALD-ZnO layer to the top OPD, resulting in the VPT operating as a non-ohmic OPD in the OFF state and as an ohmic OPD in the ON state. Benefiting from the unique transistor geometry and gate modulation capability, this hybrid VPT can achieve an *EQE* of 45,917%, a responsivity of 197 A/W, and a specific detectivity of 3.4 × 10^12^ Jones under 532 nm illumination and low drain-source voltage (*V*_ds_ = 3 V) conditions. This transistor geometry also facilitates integration with various OPDs and the miniaturization of the ZnO channel area, offering an ideal basis for the development of highly efficient VPTs and high-resolution image sensors.

## 1. Introduction

In recent years, organic vertical field-effect transistors have garnered significant attention for their potential in display [1,2,3], complementary circuit [4], nonvolatile memory [5,6], and photosensing technologies [7,8]. This vertical architecture, characterized by its short channel length, offers important advantages such as low operating voltage, high switching speed, and large aperture ratio. Two vertical transistor geometries with different source designs, namely Schottky barrier transistors and encapsulated source electrode transistors, have been developed. Schottky barrier transistors primarily utilize source electrodes that are transparent to DC electric fields, such as carbon-based or perforated conductors [9,10,11,12], placed on a dielectric to prevent shielding of the gate field and thereby modulate the Schottky barrier for current injection from the source’s top surface or sidewall. These source electrodes also provide optical transparency favored for light emission and photodetection. Several studies have demonstrated high-performance organic light-emitting transistors (LETs) and phototransistors (PTs) based on this geometry [13,14,15,16,17,18]. However, their switching performance critically relies on the careful design of the Schottky barrier and the perforated structure of the source pattern [19,20,21]. The complex manufacturing process of these source electrodes may also hinder cost-effectiveness and large-scale production. On the other hand, encapsulated source electrode transistors feature an insulator-encapsulated source embedded within the active channel [22,23,24,25,26]. This source design enables gate modulation of current injection from the source’s bottom surface while eliminating non-gated leakage, allowing for effective current control with a simple source pattern. Despite this significant advantage, fine patterning of the encapsulated source on the active channel, particularly on organic semiconductors, poses challenges in device fabrication [24]. Moreover, non-ohmic injection and low conductivity in organic channels severely impede device performance and potential applications [27,28]. Because of these issues, this transistor geometry has been employed in only a few studies of organic LETs [29,30], and has been rarely studied in PTs thus far.

Previously, we reported on encapsulated source electrode transistors that utilize an inorganic/organic hybrid scheme, where a high-mobility inorganic semiconductor serves as the channel beneath the source electrode [31]. This vertical transistor can be conceptualized as an integration of an inorganic field-effect transistor at the base and an organic diode at the top. By leveraging the inherent advantages of inorganic semiconductors, such as their compatibility with standard photolithography and resistance to organic solvents, the bottom inorganic transistor can be fabricated in various sizes to define the channel area and allows for the deposition of various organic materials on top without being restricted by their evaporation or solution processes. Furthermore, we demonstrated that the high-mobility inorganic transistor can generate an exceptionally high current density (>10 A/cm^2^), thereby achieving high-performance LETs with brightness and quantum efficiency comparable to reference OLEDs and even quantum-dot LEDs that require high driving currents [32]. Therefore, this hybrid channel scheme may address the technical challenges and performance limitations of all-organic encapsulated source electrode transistors, opening up a wide range of applications, particularly advantageous as a building block for high-resolution active-matrix panels.

In this paper, we employ this inorganic/organic hybrid channel scheme to develop vertical PT [(V)PT] based on the encapsulated source electrode transistor geometry. This is constructed by integrating an atomic-layer-deposition processed ZnO (ALD-ZnO) transistor with a prototype poly(3-hexylthiophene):[6,6]-phenyl-C_61_-butyric acid methyl ester (P3HT:PC_61_BM) blend organic photodiode (OPD) as a model system. Compared to Schottky-type organic VPTs, this hybrid VPT operates differently due to its unique source and channel design. Schottky-type organic VPTs primarily combine gate modulation capability with the function of trap-assisted photomultiplication OPDs, where the conductive channel, typically a P-type photoactive polymer, is doped with a low concentration of N-type molecules. This doping traps photoinduced electrons, facilitating the tunneling of hole current across the Schottky barrier [33,34]. On the other hand, our hybrid VPT features an ohmic contact between the source and the ALD-ZnO layer [31], and relies on the gate modulation of the ALD-ZnO transistor to control electron current. In this context, the interface properties between the ALD-ZnO and P3HT:PC_61_BM layers may play a crucial role in device performance. By conducting a comparative study of reference P3HT:PC_61_BM OPDs under various electron injection conditions, we elucidate the unique characteristics of electron injection between the ALD-ZnO and P3HT:PC_61_BM layers, as well as the impact of the ALD-ZnO layer on photogenerated holes. Furthermore, we show that the ALD-ZnO transistor can effectively modulate electron injection into the P3HT:PC_61_BM layer. These factors collectively contribute to the high performance of the VPT.

## 2. Device Fabrication and Characterization Method

**Figure 1:**
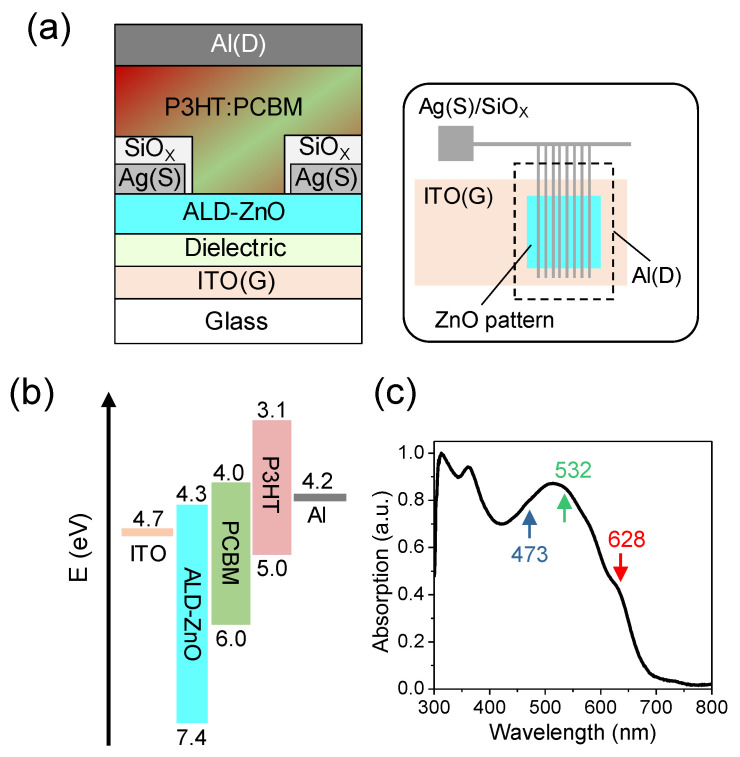
(**a**) Schematic of the P3HT:PC_61_BM VPT, constructed based on the vertical integration of an ALD-ZnO field-effect transistor and a P3HT:PC_61_BM OPD. The inset illustrates the design of the ZnO and electrode patterns. (**b**) Energy level diagram of the materials used. (**c**) Absorption spectrum of the P3HT:PC_61_BM blend film, with arrows indicating the specific wavelengths of the LEDs used for device illumination.

Figure 1a illustrates the architecture of the studied VPT. The bottom ALD-ZnO transistor was fabricated on a glass substrate with a 150-nm ITO pattern (purchased from Lumtec, with a sheet resistance of 15 Ω/sq) serving as the gate electrode. Following a 15-min ozone treatment of the cleaned ITO surface, a 15-nm Al_2_O_3_/15-nm HfO_2_ bilayer high-k dielectric was deposited via the thermal ALD technique (Ultratech Savannah,) at 80 °C, using Trimethylaluminum/Tetrakis(dimethylamido)hafnium(IV) and water as precursors. Subsequently, a 30-nm ZnO layer was deposited via ALD at 100 °C using diethylzinc and water as precursors. The ALD-ZnO layer was then patterned into a 1000 × 1000 μm^2^ square by photolithography to define the largest active region. A 40-nm Ag source electrode encapsulated with 200 nm SiO_X_ was patterned on the ALD-ZnO layer by self-aligned photolithography. This configuration and process result in a high-performance ALD-ZnO transistor with low driving voltage, minimal gate leakage, an areal capacitance of 350 nF/cm^2^, and an electron mobility of approximately 10 cm^2^/Vs. For more detailed fabrication processes and ALD-ZnO transistor characterizations, please refer to Ref. [31]. In this work, we designed a source pattern comprising eight stripes on the ZnO pattern (inset of Figure 1a), with each stripe having a width of *d* = 10 μm and a bare aperture width of *W_a_* = 100 μm between adjacent stripes. This corresponds to a geometrical aperture ratio (the ratio of the total source aperture area to the ZnO pattern) of 92%. Subsequently, a P3HT:PC_61_BM blend film was deposited on the ZnO/encapsulated source electrode patterns by spin-coating a mixed solution with a weight ratio of 1:1 in 1,2-dichlorobenzene (DCB). Both P3HT and PC_61_BM were purchased from Luminescence Technology Corp. (New Taipei, Taiwan) and used as received. The P3HT employed in this study is of the regioregular type, possessing an average molecular weight of 144 kD. The total concentrations of P3HT and PC_61_BM in the DCB solution were controlled at 30 mg/mL to achieve a film thickness of 200 nm. The P3HT:PC_61_BM blend film was then annealed at 110 °C for 10 minutes in a nitrogen glove box. Finally, the VPT was completed by evaporating an Al electrode over the ZnO pattern to serve as the drain electrode.

For comparison, we also fabricated a series of P3HT:PC_61_BM OPDs, including a standard device without ZnO, as well as devices incorporating ALD-ZnO and commercial nanoparticle ZnO (NP-ZnO) layers. All these OPDs utilize a 150-nm ITO pattern as the bottom electrode. For the ALD-ZnO device, a 30-nm ZnO layer was deposited on ITO via thermal ALD. To eliminate stray currents, the ALD-ZnO layer was patterned via photolithography to precisely cover the ITO pattern. For the NP-ZnO device, the NP-ZnO layer was prepared by spin-coating nanoparticle ink (cat. no. 807613 and 808253, Sigma-Aldrich, St. Louis, MO, USA) with work functions (WFs) of 4.3 eV and 3.9 eV, denoted as NP1-ZnO and NP2-ZnO, respectively, without further patterning. The average particle size of both NP1-ZnO and NP2-ZnO ranges from 8 to 16 nm. The thickness of the NP-ZnO layer was meticulously controlled by spin conditions to approximately 30 nm, comparable to that of the ALD-ZnO layer. Subsequently, a 200-nm P3HT:PC_61_BM blend film (1:1 weight ratio) was deposited on the ITO/ZnO pattern from DCB and then annealed at 110 °C for 10 min in a nitrogen glove box. Finally, an 80-nm Al layer was thermally evaporated to serve as the top electrode. All OPDs have dimensions of 2 × 2 mm^2^.

Figure 1b shows the energy level diagram of the ALD-ZnO, P3HT, PC_61_BM, as well as the ITO and Al electrodes. From UPS measurements, we estimated the LUMO level of the ALD-ZnO layer to be approximately 4.3 eV, which is closely aligned with the Fermi level of the NP1-ZnO layer. Figure 1c shows the absorption spectrum of the DCB-deposited P3HT:PC_61_BM film with a 1:1 weight ratio, featuring the P3HT absorption band peaking at 515 nm and the pronounced PC_61_BM absorption below 400 nm.

In this study, all optoelectronic characterizations of the OPD and VPT were conducted using an Agilent B1500A semiconductor parameter analyzer (Santa Rosa, CA, USA). Photocurrent measurements were performed by illuminating the devices from the ITO side with LEDs at specific peak wavelengths (473 nm, 532 nm, and 628 nm) for P3HT absorption, maintaining a fixed intensity of *P_in_* = 1 mW/cm^2^. The external quantum efficiency (*EQE*), responsivity (*R*), and specific detectivity (D*) were calculated according to the following equations [35]:

$$EQE\;=\frac{J_{ph}hv}{P_{in}e}=\frac{\left(J_{light}-J_{dark}\right)hv}{P_{in}e}\;\;\;$$

$$R=\frac{J_{ph}}{P_{in}}\;$$

$$\;D^{*}=\frac{R}{\sqrt{2eJ_{dark}}},$$

where *J_ph_* represents the photocurrent density, *J_light_* is the current density under illumination, *J_dark_* is the dark current density, *h* denotes the Planck constant, ν is the frequency of the incident light, and *e* is the elementary charge. The capacitance-voltage (*C*-*V*) response was measured with a 4192 A Hewlett Packard Impedance Analyzer (Palo Alto, CA, USA). The transient response was measured using an oscilloscope connected to a 50 Ohms resistor in series with the device. All electrical measurements were carried out in a nitrogen glove box. 

Additionally, the exciton generation rate *G*(z, λ), at a specific position z within the device and for an illuminated wavelength λ, was determined using the following calculation:

$$G(z,\lambda )=\frac{\lambda }{hc}Q(z,\lambda )\;$$

$$Q(z,\lambda )=\;\frac{1}{2}c\varepsilon_{0}\alpha_{\lambda }n_{\lambda }{\left|E\left(z\right)\right|}^{2}\;$$

where Q(z,λ) represents the average energy dissipated per second at position z in the material for the illuminated wavelength. Here, *c* is the vacuum speed of light, $\varepsilon_{0}$ is the vacuum permittivity, $\alpha_{\lambda }$ and $n_{\lambda }$ are the absorption coefficient and real refractive index of the material at the illuminated wavelength, respectively, and *E*(z) is the electric field distribution at position z, as simulated using Essential Macleod V12.2 software.

## 3. Results and Discussion

**Figure 2:**
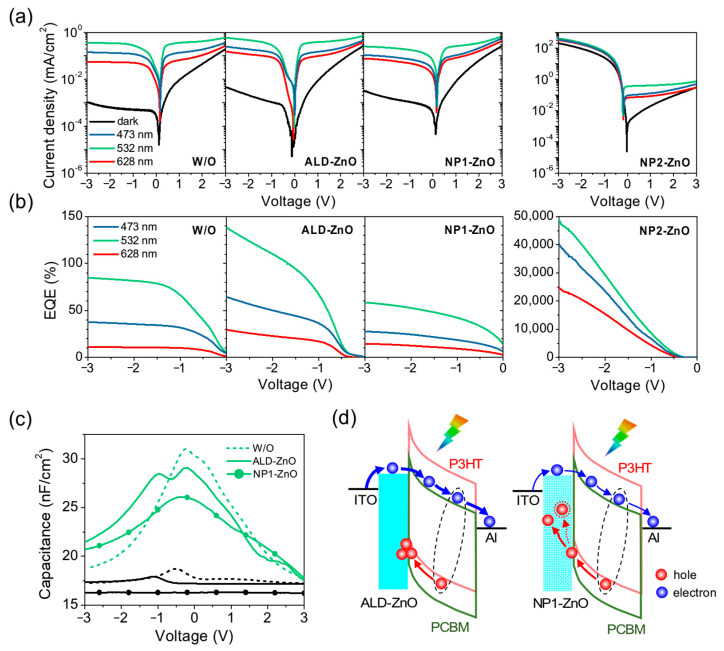
(**a**) *J*-*V* characteristics of P3HT:PC_61_BM OPDs without and with different electron injection layers (ALD-ZnO, NP1-ZnO, NP2-ZnO), measured under dark conditions and various wavelengths of illumination. (**b**) *EQE* of various OPDs under negative bias. (**c**) *C*-*V* response of the OPDs with non-ohmic injection in the dark (black line) and under 532 nm illumination (green line). (**d**) Illustration of charge injection, accumulation, and transport in the OPDs with ALD-ZnO and NP1-ZnO layers under negative bias.

In preliminary studies aimed at developing the hybrid VPT, we investigated the photoresponse properties of reference P3HT:PC_61_BM OPDs without and with different electron injection layers (ALD-ZnO, NP1-ZnO, NP2-ZnO). Figure 2a shows the *J*-*V* characteristics of these OPDs, where the ITO electrode was biased relative to the grounded Al electrode. Note that all devices exhibit similar wavelength dependence of photocurrents, peaking at 532 nm illumination, slightly decreasing at 473 nm, and being lowest at 628 nm. Consistent with theoretical expectations, this wavelength dependence of the photocurrent correlates with the exciton generation rate distribution in the P3HT:PC_61_BM layer (simulation results of the ALD-ZnO device can be seen in Supplementary Materials, Figure S1a), and somewhat corresponds with the absorption spectrum of the bare film (Figure 1c). 

Overall, the standard device without ZnO as well as those with ALD-ZnO and NP1-ZnO (WF = 4.3 eV) layers exhibit similar characteristics dominated by photocarrier dissociation behavior. Under negative bias, their *J_dark_* is suppressed below 10^−2^ mA/cm^2^, while the highest *J_light_* under 532 nm illumination tends to saturate between 10^−1^ and 1 mA/cm^2^ at −3 V, indicating non-ohmic electron injection in these OPDs. Interestingly, compared to the standard device, the ALD-ZnO device exhibits higher *J_dark_* and *J_light_*, whereas the NP1-ZnO device shows higher *J_dark_* but lower *J_light_*. As a consequence, the ALD-ZnO device achieves a maximum *EQE* of 140% under 532 nm illumination (Figure 2b), surpassing the standard device’s 85%, indicating photomultiplication due to external current injection. In contrast, the NP1-ZnO device achieves a lower *EQE* than the standard device, with a maximum value of only 58%. This result suggests that although both the ALD-ZnO and NP1-ZnO layers similarly reduce the electron injection barrier compared to the bare ITO electrode (as evidenced by their slightly higher *J_dark_*), the structure of the ZnO layer may have additional effects on the photocurrent, which we will discuss further below. On the other hand, the NP2-ZnO (WF = 3.9 eV) OPD behaves like an electron-only diode with ohmic contact under negative bias, exhibiting a rapid increase in both *J_dark_* and *J_light_* to over 200 mA/cm^2^ at −3 V. This current level is several orders of magnitude higher than that of other OPDs and may reflect the intrinsic electron conductivity of the P3HT:PC_61_BM layer. Due to the highly efficient electron injection, the NP2-ZnO device can achieve a maximum *EQE* of 48,947% at −3 V (Figure 2b). 

The derived values of *R* and D* for all studied OPDs, along with their measured transient response under 532 nm illumination, are provided in Supplementary Materials, Figure S2. For clarity, the characteristics of all OPDs under 532 nm illumination and −3 V bias are summarized in Table 1. It can be observed that, while non-ohmic and ohmic devices show several orders of magnitude difference in *EQE* and *R*, they possess similar D* values in the range of 10^11^ to 10^12^ Jones, and similarly short rise and fall times of <0.1 ms.

To gain deeper insights into the influence of the ZnO layer structure on photocurrents, we measured the *C*-*V* response of the ALD-ZnO OPD and compared it with standard and NP1-ZnO devices (non-ohmic case) under 532 nm illumination. As shown in Figure 2c, the capacitance of all devices increases upon illumination, peaking around zero bias and decreasing with higher bias. This clearly reveals the phenomenon of photocarrier dissociation in these devices. At low bias, a large number of dissociated photocarriers remain in the device, resulting in high capacitance. As the bias increases positively or negatively, the photocarriers are gradually extracted, causing the capacitance to decrease. Interestingly, the ALD-ZnO device exhibits not only a primary peak at 0 V but also a secondary peak at −1 V under illumination, indicating the presence of charge accumulation. By contrast, the NP1-ZnO device shows a monotonic decrease in capacitance at negative bias, similar to the standard device. Based on the *C*-*V* response results, we can clarify the different effects of the ALD-ZnO and NP1-ZnO layers on charge injection, accumulation, and transport in the P3HT:PC_61_BM OPD under negative bias, as illustrated in Figure 2d. In the ALD-ZnO device, photogenerated holes migrate towards the ITO electrode but are blocked by the ALD-ZnO layer, resulting in significant accumulation at the ZnO/P3HT:PC_61_BM interface. This accumulation may generate a positive electric field, which, combined with the higher-lying LUMO of ALD-ZnO relative to the ITO WF, enhances the injection of external electron current and accounts for an *EQE* exceeding 100%. On the other hand, the NP1-ZnO device behaves similarly to the standard device and shows no hole accumulation under negative bias, possibly due to the porous structure of the nanoparticles. Although NP1-ZnO has a lower WF compared to ITO, photogenerated holes may leak through the nanoparticle layer and become trapped by defects, resulting in an even lower *EQE* than the standard device. From the transient response measurements, it can be found that the rise and fall times of the ALD-ZnO OPD are slightly longer than those of the standard and NP1-ZnO devices (see Table 1), which may be a consequence of hole accumulation within the device.

Putting all the P3HT:PC_61_BM OPD data together, we demonstrate the critical role of electron injection in device performance and distinguish between non-ohmic and ohmic injection devices. Specifically, incorporating an ALD-ZnO layer between the ITO cathode and the P3HT:PC_61_BM layer slightly reduces the electron injection barrier, though the injection remains non-ohmic. However, compared to the NP-ZnO layer, the ALD-ZnO layer can effectively block and accumulate photogenerated holes, which facilitates efficient electron injection under illumination and can even achieve photomultiplication with an *EQE* exceeding 100%.

**Figure 3:**
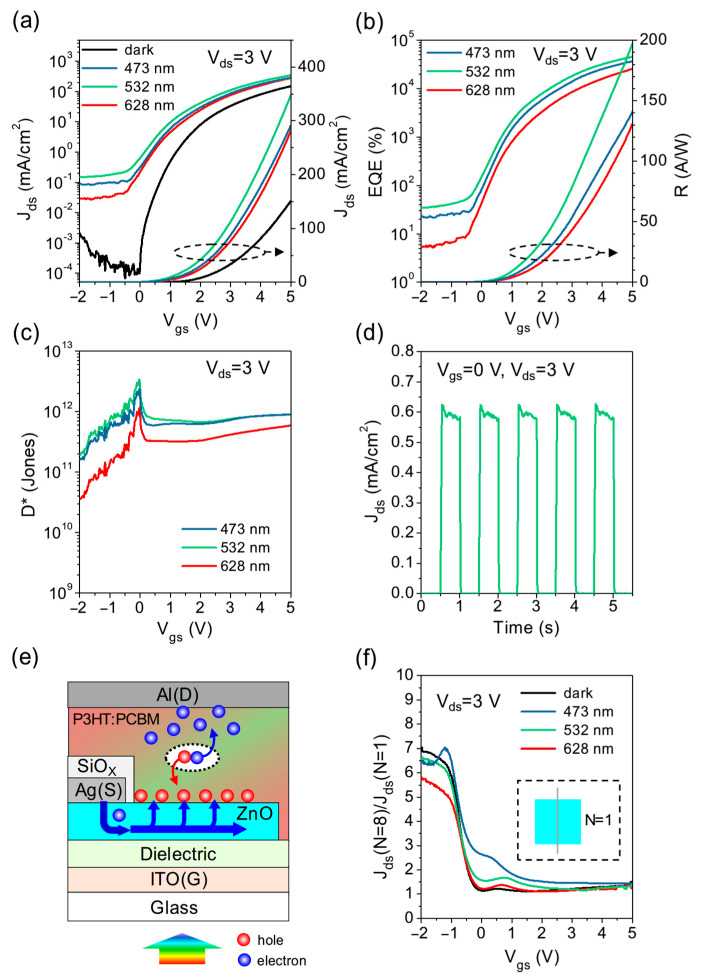
(**a**) Transfer characteristics of the VPT with P3HT:PC_61_BM OPD integrated on an ALD-ZnO transistor, measured at *V*_ds_ = 3 V. (**b**) *EQE*, *R*, and (**c**) D* of the VPT. (**d**) Transient response of the VPT at *V*_gs_ = 0 V and *V*_ds_ = 3 V, measured under 532 nm illumination. (**e**) Illustration of the operation mechanism of the VPT under illumination. (**f**) Ratios of *J_dark_* and *J_light_* of VPTs designed with eight source stripes (*N* = 8) and a single stripe (*N* = 1) positioned at the center of the ZnO pattern.

Next, we investigate the VPT by integrating the P3HT:PC_61_BM OPD on the ALD-ZnO transistor. Figure 3a shows the transfer characteristics of the VPT under dark and illuminated conditions at different wavelengths, measured at *V*_ds_ = 3 V for direct comparison with the OPDs biased at −3 V. This VPT, driven by the ZnO transistor, clearly exhibits n-type transistor behavior. Despite the complex current distribution within the VPT [36], we simplify the device analysis by considering the entire source aperture on ZnO as the effective channel area to evaluate the average current density. In the dark, the VPT exhibits a low OFF-current density of approximately 10^−4^ mA/cm^2^ due to effective suppression by the SiO_X_ source encapsulation, and it switches on at *V*_gs_~0 V, achieving a maximum ON-current density of 151 mA/cm^2^ at *V*_gs_ = 5 V. When illuminated at various wavelengths, the current density increases across the entire *V*_gs_ regime, with a particularly notable enhancement of 2–3 orders of magnitude in the OFF state (*V*_gs_ < 0 V). Overall, the wavelength-dependent response closely mirrors that of the OPDs, with the highest photocurrent obtained at 532 nm, followed by 473 nm, and the lowest at 628 nm. This correlation can be verified by the similar exciton generation rate distributions in the P3HT:PC_61_BM layer of both the VPT and the reference OPD with an ALD-ZnO layer at various wavelengths (see Supplementary Materials, Figure S1b). Figure 3b,c show the calculated *EQE*, *R*, and D* of the VPT. Under 532 nm illumination, the VPT achieves a maximum *EQE* of 45,917% and a maximum *R* of 197 A/W, corresponding to the highest *J_ph_* of 197 mA/cm^2^ at *V*_gs_ = 5 V. On the other hand, a maximum D* of 3.4 × 10^12^ Jones is obtained at *V*_gs_~0 V, surpassing all the P3HT:PC_61_BM OPDs, which can be attributed to the relatively low *J_dark_* in the OFF state. The characteristics of the VPT under 532 nm illumination are summarized in Table 1.

Based on the previous study of P3HT:PC_61_BM OPDs, we can further interpret the operation mechanism in the VPT, as illustrated in Figure 3e. Under illumination, photogenerated electrons and holes are driven by the vertical drain field to migrate upward and downward, respectively. As demonstrated in the ALD-ZnO OPD, photogenerated holes may accumulate on the top surface of the ALD-ZnO layer, creating a positive electric field that facilitates electron injection into the P3HT:PC_61_BM layer. Such photoinduced hole accumulation may even extract electrons directly from the source edge, resulting in a significant increase in the photocurrent in the OFF state. Furthermore, it is worth noting that at *V*_gs_~0 V, the VPT exhibits *J_dark_* and *J_light_* levels comparable to those of OPDs with non-ohmic injection. At higher *V*_gs_, both currents increase by several orders of magnitude, approaching the levels observed in the NP2-ZnO OPD with ohmic injection. These comparative characteristics suggest that the gate field can effectively modulate electron injection into the top OPD. According to Sawatzki’s theory and our previous studies [31,32,36], the current paths in this vertical transistor geometry involve a complex balance between lateral diffusion and vertical drift, forming a lateral electron density gradient within the source aperture (i.e., channel depth) influenced by the stacked materials and the applied voltages. Given that ALD-ZnO has significantly higher electron mobility and a lower LUMO level relative to PC_61_BM, electrons in the ALD-ZnO layer may tend to undergo long-distance lateral diffusion and accumulate near the top ZnO surface before being driven into the P3HT:PC_61_BM layer. As *V*_gs_ increases while *V*_ds_ remains fixed, not only are more electrons injected from the source, but the channel depth also expands [36], resulting in a higher density and larger area of electron accumulation near the top ZnO surface. Consequently, this overcomes the electron injection barrier and leads to a significant increase in current with *V*_gs_. The photocurrent in the ON state can be further enhanced by photoinduced hole accumulation on the ZnO surface. However, this hole accumulation effect on electron injection is less pronounced than the gating effect in the ON state, so the enhancement of the photocurrent is not as evident as in the OFF state.

Although the length scale of “channel depth” in a non-emitting VPT is difficult to resolve experimentally and requires detailed simulation, its impact on electron injection properties and device performance can be evidenced through different source designs. Figure 3f compares the current density of VPTs designed with eight source stripes (*N* = 8) and a single stripe (*N* = 1) positioned at the center of the ZnO pattern. It can be observed that both *J_dark_* and *J_light_* in the OFF state exhibit an approximately proportional increase with the number of source stripes *N*, while the current density in the ON state shows a weak dependence on *N*. This result manifests the different electron injection properties between the ALD-ZnO and P3HT:PC_61_BM layers in the OFF and ON states. Due to non-ohmic injection and a narrow channel depth in the OFF state, using multiple source stripes can increase the injection area and hence the current density. However, since the injection becomes nearly ohmic and the channel depth is significantly broadened in the ON state, a single stripe is capable of producing a high current density approaching that of multiple stripes. In this work, we optimized the source design with eight source stripes mainly to improve the detectivity (∝ *J_ph_* × *J_dark_*^−1/2^) in the OFF state, while ensuring a high ON-current and a large aperture ratio of over 90%.

Figure 3d shows the transient response of the photocurrent of the VPT under 532 nm illumination in the OFF state (*V*_gs_ = 0 V), with rise and fall times estimated to be 28 ms and 17 ms, respectively (Table 1). These times are slightly reduced to 8 ms and 7.8 ms in the ON state (*V*_gs_ = 5 V), possibly due to increased electron conductivity in ZnO and more efficient injection into the P3HT:PC_61_BM layer. Compared to all OPDs, the slower response in the VPT may stem from the micrometer-scale SiO_X_ encapsulation around the source edge, which creates a longer path for electrons to pass through the ZnO to the P3HT:PC_61_BM layer. Nevertheless, the photocurrent shows sufficient stability with little decrease under long-term periodic photoexcitation, indicating the absence of continuous accumulation or dissipation of photogenerated charges within the device. We anticipate that further optimization of the source pattern and encapsulation geometry may lead to improved device performance and a shorter response time.

## 4. Conclusions

We have developed a high-performance VPT by integrating an ALD-ZnO transistor with a P3HT:PC_61_BM OPD based on an encapsulated source electrode geometry, and have provided clear insights into the device’s mechanisms. From preliminary studies of reference OPDs without and with various ZnO electron injection layers, we have elucidated the role of controlling electron injection on device performance and confirm the non-ohmic electron injection between the ALD-ZnO and P3HT:PC_61_BM layers. Importantly, the ALD-ZnO layer demonstrates the ability to block and accumulate photogenerated holes, a phenomenon absent in the NP-ZnO layer. This accumulation of photogenerated holes enhances electron injection, which contributes to efficient photoresponse in both the OPD and VPT. Furthermore, we have demonstrated that the gate field of the VPT effectively modulates electron injection from the ALD-ZnO layer into the top OPD, enabling the VPT to function as a non-ohmic OPD in the OFF state and as an ohmic OPD in the ON state. Under 532 nm illumination and low *V*_ds_ (3 V) conditions, the VPT achieves a maximum D* of 3.4 × 10^12^ Jones in the OFF state, benefiting from its low dark current. In the ON state with a high photocurrent, it can reach maximum values of an *EQE* of 45,917% and an *R* of 197 A/W. This transistor geometry also offers significant advantages for integrating with various OPDs and miniaturizing the ZnO channel area, promising further enhancements in VPT properties and applications in high-resolution image sensors.

## Figures and Tables

**Table 1:** Summary of the characteristics of various P3HT:PC_61_BM OPDs and VPT under 532 nm illumination, with the OPDs biased at −3 V and VPT biased at *V*_ds_ = 3 V for comparison.

		J_dark_/J_light_ (mA/cm^2^)	EQE (%)	R (A/W)	D* (Jones)	Rise/Fall Time (ms)
OPD	W/O	0.0011/0.36	85	0.36	6.1 × 10^11^	0.074/0.058
	ALD-ZnO	0.0047/0.59	138	0.59	4.8 × 10^11^	0.098/0.09
	NP1-ZnO	0.0033/0.25	58	0.25	2.4 × 10^11^	0.043/0.032
	NP2-ZnO	204/414	48,947	210	8.2 × 10^11^	0.068/0.051
VPT	OFF (*V*_gs_ = 0 V)	0.00012/0.64	149	0.64	3.4 × 10^12^	28/17
	ON (*V*_gs_ = 5 V)	151/348	45,917	197	9.0 × 10^11^	8/7.8

## Data Availability

The original contributions presented in this study are included in the article. Further inquiries can be directed to the corresponding author.

## Supplementary Materials

The following supporting information can be downloaded at: https://www.mdpi.com/article/10.3390/mi15111397/s1, Figure S1: Exciton generation rate distribution in the P3HT:PC_61_BM layer of the OPD with an ALD-ZnO layer and the VPT; Figure S2: Responsivity, specific detectivity, and transient response of all P3HT:PC_61_BM OPDs.
