# Supplementary material for: Study of Vertical Phototransistors Based on Integration of Inorganic Transistors and Organic Photodiodes
Source: Micromachines (Basel). 2024 Nov 20;15(11):1397. doi: 10.3390/mi15111397 (PMC11596657; doi:10.3390/mi15111397)
Supplement: Supplementary file 1 [file micromachines-15-01397-s001.zip › micromachines-3314775-supplementary.pdf]

Supplementary Information

Study of Vertical Phototransistors Based on Integration of Inorganic Transistors and Organic Photodiodes

Jui-Fen Chang *, Ying-You Lin and Yu-Ming Li

Department of Optics and Photonics, National Central University, Zhongli 320317, Taiwan; l.y.y111226061@gmail.com (Y.-Y.L.); james80779771@gmail.com (Y.-M.L.)

* Correspondence: jfchang@dop.ncu.edu.tw

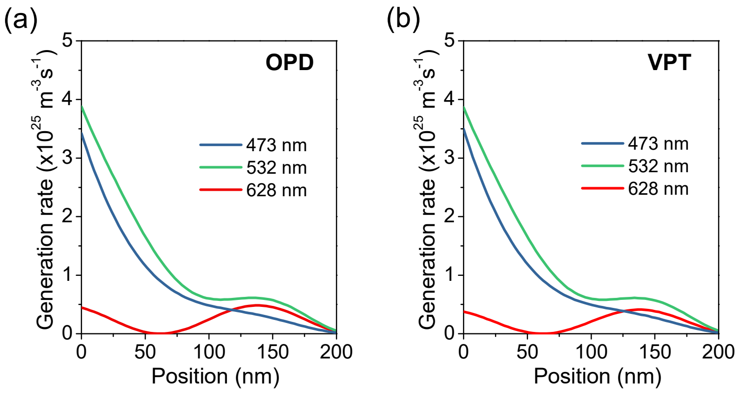

Figure S1. Calculated exciton generation rate distribution at various illuminated wavelengths in the P3HT:$PC_{61}BM$ layer of (a) the OPD with an ALD-ZnO layer and (b) the VPT. The interface between the ALD-ZnO and P3HT:$PC_{61}BM$ layers is positioned at zero. The P3HT:$PC_{61}BM$ layer has a thickness of 200 nm.

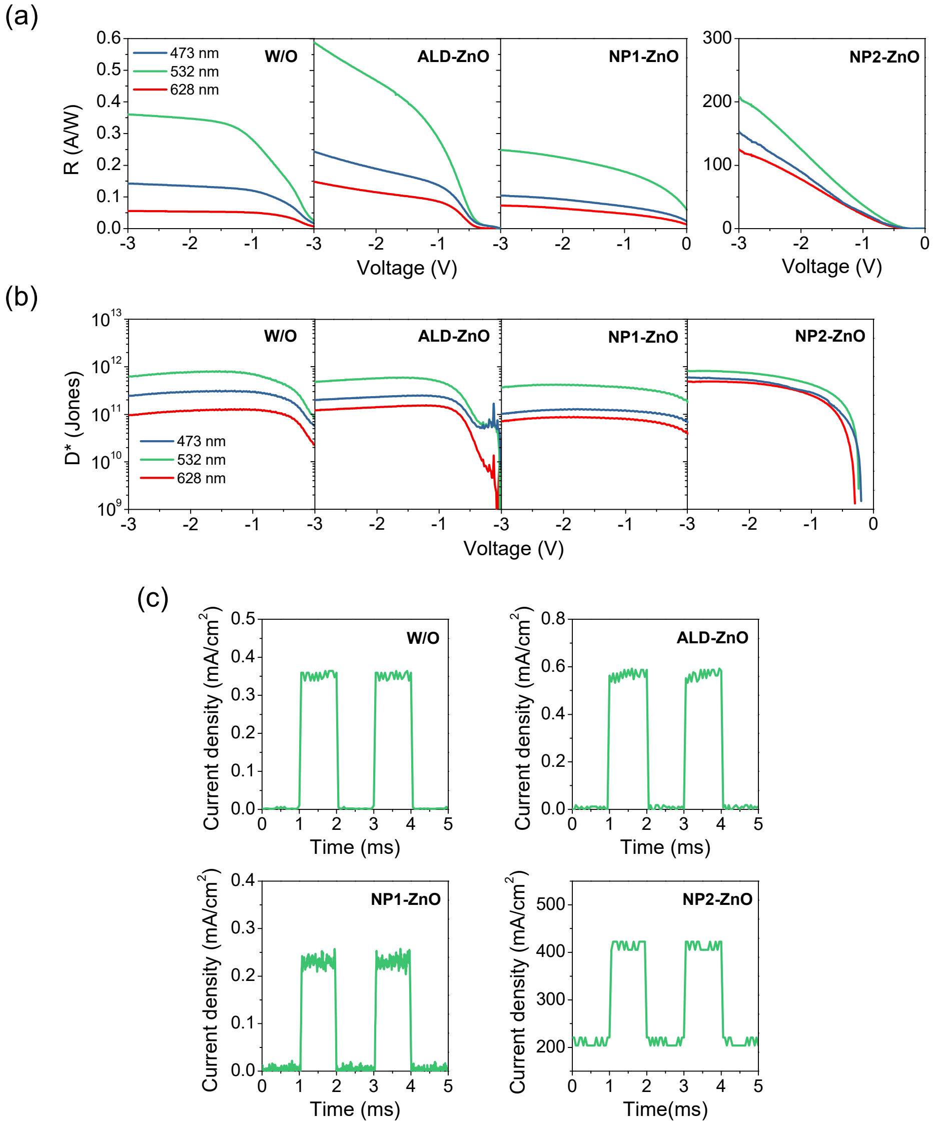

Figure S2. (a) Responsivity, (b) specific detectivity, and (c) transient response of P3HT:$PC_{61}BM$ OPDs without and with different electron injection layers (ALD-ZnO, NP1-ZnO, NP2-ZnO). The transient response was measured under 532 nm illumination.
